# Assessment of Antibiotic Pharmacokinetics, Molecular Biomarkers and Clinical Status in Critically Ill Adults Diagnosed with Community-Acquired Pneumonia and Receiving Intravenous Piperacillin/Tazobactam and Hydrocortisone over the First Five Days of Intensive Care: An Observational Study (STROBE Compliant)

**DOI:** 10.3390/jcm11144140

**Published:** 2022-07-16

**Authors:** István Vincze, Rita Czermann, Zsuzsanna Nagy, Mária Kovács, Michael Neely, Róbert Farkas, Ibolya Kocsis, Gellért Balázs Karvaly, Csaba Kopitkó

**Affiliations:** 1 Department of Laboratory Medicine, Semmelweis University, H-1083 Budapest, Hungary; farkas.robert@med.semmelweis-univ.hu (R.F.); koc-sis.ibolya@med.semmelweis-univ.hu (I.K.); karvaly.gellert_balazs@med.semmelweis-univ.hu (G.B.K.); 2 Central Department of Anaesthesiology and Intensive Care, Uzsoki Teaching Hospital, H-1145 Budapest, Hungary; czermann@gmail.com (R.C.); kopcsab2@gmail.com (C.K.); 3 Central Department of Laboratory Diagnostics, Uzsoki Teaching Hospital, H-1145 Budapest, Hungary; nagy.zsuzsanna@uzsoki.hu (Z.N.); kovacsm@uzsoki.hu (M.K.); 4 Laboratory of Applied Pharmacokinetics and Bioinformatics, Children’s Hospital of Los Angeles, Keck School of Medicine, University of Southern California, Los Angeles, CA 90033, USA; mneely@chla.usc.edu

**Keywords:** piperacillin, tazobactam, community acquired pneumonia, intensive care, hydrocortisone, cytokine storm, population pharmacokinetics, therapeutic drug monitoring

## Abstract

Severe community-acquired pneumonia (CAP) is a condition that frequently requires intensive care and, eventually, can cause to death. Piperacillin/tazobactam antibiotic therapy is employed as an empiric intravenous regimen, in many cases supplemented with intravenous bolus hydrocortisone treatment. The individual and condition-dependent pharmacokinetic properties of these drugs may lead to therapeutic failure. The impact of systemic inflammation, as well as of hydrocortisone on the altered pharmacokinetics of piperacillin is largely unknown. The protocol of a clinical study aimed at the characterization of the pharmacokinetics of piperacillin and tazobactam and its association with the concentrations of inflammatory markers and adrenal steroids during CAP therapy will be investigated in up to 40 critically ill patients. The serum concentrations of piperacillin and tazobactam, cortisol, cortisone, corticosterone and 11-deoxycortisol and interleukin-6 levels, as well as routine clinical chemistry and hematology parameters will be monitored from the beginning of treatment for up to five days. Nonparametric population pharmacokinetic modeling and Monte-Carlo simulations will be performed to make estimates of the pharmacokinetics of piperacillin and tazobactam and the probability of pharmacokinetic-pharmacodynamic target attainment. The observed individual characteristics and changes will be correlated with clinical and laboratory findings. The protocol of the observational study will be designed following the STROBE guideline.

## 1. Introduction

### 1.1. Background

Severe community-acquired pneumonia (CAP) is a leading cause of death among infectious diseases. Up to 20% of adults diagnosed and hospitalized with CAP eventually require intensive care. Of the CAP patients treated at the intensive care unit (ICU), 40–80% require mechanical ventilation, approximately 50% develop severe sepsis or septic shock and the overall mortality is 20–50% [1,2,3]. Therefore, research to improve the outcomes and the efficacy of the intensive therapy of these patients is of crucial importance.

CAP patients treated in the ICU receive complex parenteral therapy from the first day. The most important components are (1) antibiotics, with piperacillin being commonly employed along with the β-lactamase inhibitor tazobactam, and (2) a steroid, such as hydrocortisone (identical to cortisol) [4]. Little is known about the plasma and tissue drug exposure among critically ill CAP patients with often altered pharmacokinetics, especially in association with the resulting changes in the serum levels of adrenal steroids.

Additionally, the characteristic of the acute inflammatory phase may also be related to treatment measures since the optimal elimination of the infection requires an adequate initial response of immune system that considers the time and level of extension. Mortality correlates significantly with the magnitude of inflammatory response occurring in CAP, characterized by elevated concentrations of IL-6 and IL-10 [5]. Hydrocortisone effectively reduces this exaggerated response, termed as cytokine storm [6]. Several publications support intravenous hydrocortisone in septic shock [7,8,9], yet its impact on related laboratory parameters including IL-6 and IL-10, and its association with altered pharmacokinetic values of piperacillin and tazobactam, is not completely clear.

Piperacillin is a β-lactam agent effective against several Gram-positive and Gram-negative aerobic and anaerobic strains. It is used in combination with tazobactam, which increases the efficacy of piperacillin and widens its antibiotic spectrum [10]. However, the relationship between its pharmacokinetic properties with therapeutic failure is unclear. Both drugs are excreted primarily without transformation through the kidneys; therefore, changes in renal function may alter their elimination [11]. Protein binding of piperacillin and tazobactam was found to be about 30% [12]; the unbound concentration of piperacillin and tazobactam can be simulated based on this value. The bacteriostatic/bactericidal and resistance suppression efficacy of piperacillin is frequently estimated by calculating its pharmacokinetic/pharmacodynamic (PK/PD) index to establish a direct mathematical relationship between the duration (t) for which piperacillin attains free concentrations (f) higher than the minimal inhibitory concentration (MIC). ft>MIC of 40–50% of the dosing interval is often targeted as a general measure. Optimally, however, PK/PD targets, e.g., steady-state free piperacillin concentrations attaining fivefold MIC, should be set with the aim of attaining bactericidal levels in each dosing cycle [13,14,15,16]. Population pharmacokinetic modelling followed by Monte-Carlo simulation and the estimation of the probability target attainment (PTA) are concepts proposed for application by leading organizations addressing international recommendations such as the European Society of Clinical Microbiology and Infectious Diseases (EUCAST) and the European Medicines Agency (EMA) [17,18]. Therapeutic drug monitoring (TDM) plays an important role in abovementioned clinical decision support. Recently, however, TDM of piperacillin/tazobactam therapy at ICU did not significantly improve the clinical outcome of treatment comparison to fixed dose administration [19].

Information on the relationship between piperacillin/tazobactam pharmacokinetics, the adrenocortical function and the clinical status is essential for conducting the personalized therapeutic regime of severe CAP patients at the ICU, which is possibly the key to improving treatment outcomes. We found no clinical studies conducted with the perspective of the complex monitoring of critically ill CAP patients by the simultaneous characterization of antibiotic pharmacokinetics, adrenocortical function, the general clinical status, and routine laboratory parameters. In the current study, we will investigate these relationships to promote the individualization of future treatments.

The study protocol was written by the STROBE guideline [20], thus it is expected to support clinical research to be scientifically sound, feasible, accurate, reproducible and comparable with results originated from a similar clinical analysis. STROBE checklist of the study protocol is presented in the Supplementary Materials.

### 1.2. Objectives of the Observational Study

The overall aim of this study is to explore the potential relationships between exposure to piperacillin and tazobactam, the circulating concentrations of adrenal steroids following the administration of hydrocortisone, the activity of the immune system and the clinical status of patients treated with severe CAP (including those developing an acute exacerbation of chronic obstructive pulmonary disease) at the ICU [21]. We will investigate the associations between the pharmacokinetic properties of the studied antibiotics, the concentrations of various adrenal steroids as well as further clinical and laboratory parameters.

We sought to accomplish the following objectives:

The **overall objective** of the study was to establish a methodology for the evaluation of the consequences of pharmacological intervention as part of the initial phase of the intensive care of CAP patients with the perspective of optimizing treatment outcomes.

**Objective 1** is to establish population pharmacokinetic models of piperacillin and tazobactam in critically ill adult patients diagnosed with severe CAP and treated with an intravenous piperacillin/tazobactam preparation, as well as to monitor intra-individual changes in the pharmacokinetic properties within each subject over the first five days of treatment. Additionally, we plan to monitor the changes in concentrations of endogenous steroids during the five-day of treatment and evaluate the possible relationship with pharmacokinetic parameters of piperacillin and tazobactam.

**Objective 2** is to investigate the chance of successful microbiological cure by performing simulations based on the established population pharmacokinetic models.

**Objective 3** is to evaluate the concentrations of endogenous molecular biomarkers (glucocorticoids and inflammatory markers), which are supposed to be appropriate to characterize clinical status and to relate them to changes in PK parameters of piperacillin and tazobactam.

High inter- and intra-individual variability of PK parameters of piperacillin and tazobactam pharmacotherapy is expected in critically ill patient treated in ICU, which is related to the outcome of treatment.

The goal of this study is to test and evaluate this concept by developing a nonparametric population pharmacokinetic model of piperacillin and tazobactam in these specific populations and by comparing the observed changes in the concentration time curves to other clinical, physiological and laboratory parameters.

The results of this hypothesis-generating study are expected to provide a better understanding of the relationships between antibiotic pharmacokinetics, hormonal status and the clinical outcome of CAP patients treated at the ICU.

## 2. Methods and Analysis

### 2.1. Observational Study Design

**Figure 1:**
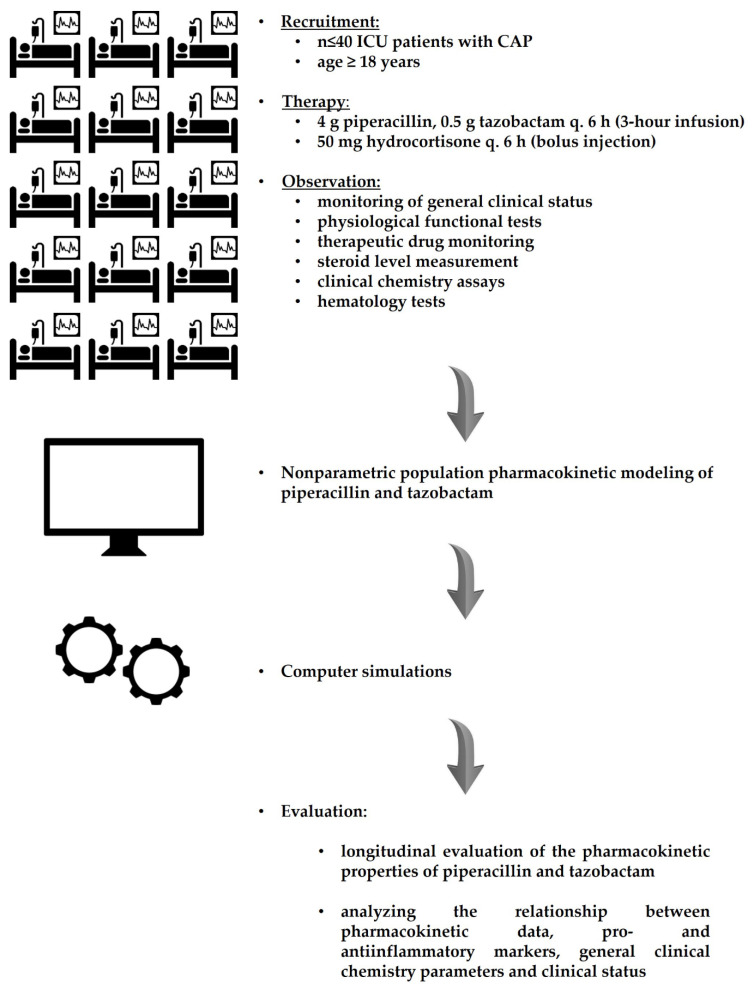
Overview of the study design.

This was a prospective, nonrandomized, noninterventional, unicentric and observational cohort study. Patients who meet the eligibility criteria are to be recruited between 1 September 2022 and 15 April 2024 (1.5-year period). The involvement of subjects will begin upon their admission to the Department of Anesthesiology and Intensive Care, Uzsoki Teaching Hospital, and will continue until an exit event occurs, or will be discontinued after day 5. The overview of the study design is presented in Figure 1.

### 2.2. Participants, Eligibility Criteria and Exit Events

We will recruit up to 40 adult patients (age ≥ 18 years) diagnosed with severe CAP and receiving piperacillin/tazobactam and hydrocortisone based on the treatment policy effective at the Central Department of Anesthesiology and Intensive Care, Uzsoki Teaching Hospital Diagnosis. The respective treatment policy has been established based on the international American Thoracic Society and the Infectious Diseases Society of America (ATS/IDSA) 2019 guidelines, according to which the validated definition of severe CAP includes either one major criterion or three minor criteria (Table 1) [22,23].

The inclusion criteria is established as patients with consideration of community-acquired pneumonia clinically suspected to be caused by infectious disease of *Pseudomonas aeruginosa*. The following risk factors of *Pseudomonas aeruginosa* infection are taken into consideration during diagnosis: chronic obstructive pulmonary disease, chronically persistent home ventilation therapy, status of diabetes mellitus, proven alcohol use disorder, long-term steroid therapy and continuing pharmacotherapy using immunosupressants. Applied antimicrobial prophylaxis before community-acquired pneumonia and registered hospitalization in three months are also connected to clinically relevant assumption to *Pseudomonas aeruginosa* infection [24].

Due to the severe condition of the subjects, they will not be approached directly, but the informed consent of close relatives who are legally capable of acting on behalf of the patients will be requested.

The study period will be terminated immediately before day 5 concerning subjects who experience any of the following events (i.e., exit events): (1) death; (2) stage 5 kidney disease requiring renal replacement therapy; (3) discharge from ICU as a result of a remarkable improvement in the subject’s clinical condition; or (4) any other medical events resulting in a special condition that, directly or due to measures regarding its treatment, have an impact on the treatment protocol, on the clinical response to treatment or on the pharmacokinetic properties of piperacillin, tazobactam or hydrocortisone. Data collected before an exit event occurs will be employed for evaluation.

Required renal replacement therapy, age < 18 years, state of pregnancy or breast feeding, as well as known intolerance for specified drug components, are defined as applicable exclusion criteria.

Subjects who withdraw their consent to participate will also be excluded immediately without any negative impact to their care or other services.

The decision to terminate the investigation of a subject (along with keeping his/her records and by including the data obtained in the evaluation of the research) or to exclude a subject will be made by the principal investigator (Cs. K.).

### 2.3. Protocol of Treatment

Based on the therapeutic indication, enrolled subjects will be administered 3-h intravenous infusion containing 4 g piperacillin along with 0.5 g tazobactam every 6 h (q6h). No bolus dose of piperacillin/tazobactam will be administered at the beginning of treatment. Intravenous injection of 50 mg hydrocortisone will be applied q6h. Other therapeutic measures will be applied according to effective international and local protocols guiding the treatment of severe CAP patients at the ICU.

### 2.4. Sample Collection

Blood and tracheal secretion will be collected from subjects. Blood samples will be drawn via the cannula inserted into the vena cava superior strictly based on the relevant therapeutic protocols. The issue corresponding to contamination of the samples with flushing liquid (cannula used for sampling of venous blood is continuously rinsed by 0.9% isotonic saline) is addressed by discarding 3 mL of solution (mixture of blood and flushing liquid) before blood sample is collected for investigation.

While the femoral vein could also be accessed, its location (i.e., below the plane of the entry of the hepatic veins into the circulation) may result in samples which would be different concerning their composition. No risks other than those associated with blood sampling employed routinely as part of the provided intensive care have been identified. Tracheal secretions will be collected through the endotracheal tube by inserting a suction catheter connected to a sputum trap (Medicoplast GmBH, Illingen, Germany).

**Figure 2:**
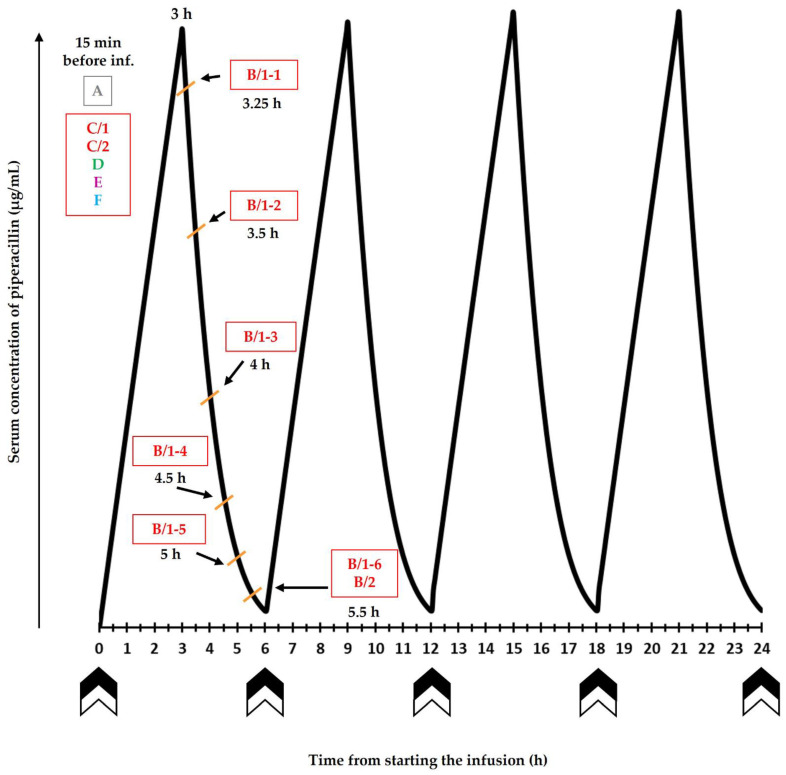
Protocol of sampling showing the first 24 h of treatment with piperacillin/tazobactam and with hydrocortisone. The same protocol is applied up to 5 days following admission to the intensive care unit. Drug administration (piperacillin/tazobactam and hydrocortisone) is indicated by double arrow (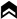). Sample and aliquot identifiers are displayed in colored boxes.

The planned sampling scheme is shown in Figure 2. The laboratory methodologies to be employed are listed in Table 2. 

#### 2.4.1. Sample ‘A’: Tracheal Secretions

The susceptibility of the pathogen CAP to piperacillin will be evaluated by collecting tracheal secretions before starting the antibiotic treatment, and assuming that no exit event occurs, on day 4 of the treatment, and by establishing the MIC of piperacillin/tazobactam.

#### 2.4.2. E-Test Method of Antibiotic Susceptibility, MIC Value

A series (15) of two-fold dilutions of an antibiotic are incorporated on a plastic carrier strip (bioMérieux Inc., Durham, NC, USA). From the strip antibiotic agent could diffuse freely into the Mueller–Hinton agar to generate a diffusion gradient along the length of the strip. After incubation the MIC could be defined as the point where the growth inhibition ellipse intersects the MIC scale on the strip. Turnaround time of the measurement could differ depending on the patient specific clinical status but generally it is conducted in 48 h [25].

#### 2.4.3. Samples ‘B/1-1′–‘B/1-6′: Native Blood Sample

To evaluate the serum concentrations of piperacillin and tazobactam, 6 native venous blood samples with a volume of up to 3.5 mL will be collected into vacutainer tubes on each of the first 5 days of antibiotic treatment at the following relative times with respect to the initiation of the daily first intravenous piperacillin/tazobactam infusion: 3.25 h; 3.5 h; 4 h; 4.5 h; 5 h; and 5.5 h. Following their collection, samples will be centrifuged immediately. Separated serum will be transported in a 2.0-mL polypropylene microcentrifuge tube for further processing to the Central Laboratory of Uzsoki Teaching Hospital.

Aliquots B/1-1–B/1-6 (piperacillin, tazobactam): In total, 100 µL serum will be separated from samples ‘B/1-1′–‘B/1-6′ and 20 µL Chromsystems^®^ Antibiotics with HPLC Priming Solution (cat. 61012) will be added. The mixture will be vortexed and stored at −70 °C until analysis. Serum concentrations of piperacillin and tazobactam will be measured using high performance liquid chromatography with absorbance detection (HPLC-UV) using Chromsystems^®^ Antibiotics in serum/plasma with HPLC reagent kit with a CE-IVD certificate (ABL&E-Jasco Hungary Ltd., Budapest, Hungary) [26]. Total concentrations of piperacillin and tazobactam are measured by Chromsystems^®^ kit. The protein binding was found to be about 30% for both drugs [12] and this value will be applied for assessment of unbound concentration. A maximum of 30 aliquots B/1 will be analyzed for each subject. Samples will be stored at −70 °C for 1 month following analysis.

Aliquot B/2 (cortisol, cortisone, corticosterone, 11-deoxycortisol): In total, 250 µL serum will be separated from sample ‘B/1-6′ and stored at −70 °C until analysis. These aliquots will be prepared from the 5.5-h samples collected after the initiation of the first piperacillin/tazobactam infusion on each day. The daily first infusion of piperacillin/tazobactam will be applied between 7.00–9.00 a.m., and aliquot ‘B/2′ will be obtained in a 2-h interval (12.30–2.30 p.m.). Consequently, the circadian rhythm of the molecules of interest is expected not to have an appreciable impact on the results.

The aliquots will be stored at −70 °C until analysis. Up to 5 Aliquots B/2 will be collected from each subject. Cortisol, cortisone, corticosterone and 11-deoxycortisol will be assayed using ultra-high performance liquid chromatography and tandem mass spectrometry (LC-MS/MS) using a validated in-house method [27]. Samples will be stored at −70 °C for 1 year following analysis.

#### 2.4.4. Sample ‘C’: Native Blood

Here, 1 tube (8 mL) of venous native blood will be collected to perform interleukin-6 (IL-6) and routine clinical chemistry assays on each day of treatment prior to the administration of the first piperacillin/tazobactam infusion. Samples will be centrifuged within 2 h. A maximum of 5 of these samples are going to be donated by each subject.

Aliquot C/1 (IL-6): In total, 1 mL serum will be separated and stored at −18 °C until analysis. using a Roche Cobas e601 analyzer with Roche Cobas Elecsys IL-6 reagent (Roche Hungary Ltd., Budapest, Hungary). Samples will be stored at −70 °C for 1 year following analysis.

Aliquot C/2 (procalcitonin, bilirubin, glucose, potassium, creatinine, sodium, urea, albumin): In total, 3 mL serum will be separated and assayed on the same day using a Roche Cobas Elecsys Brahms platform for assaying procalcitonin. The other tests (bilirubin, glucose, potassium, creatinine, sodium, urea and albumin) will be performed on a Beckman Coulter analyzer (Beckman Coulter Hungary Ltd., Budapest, Hungary). Samples will be stored at 2–8 °C for 2 days following analysis.

#### 2.4.5. Sample ‘D’: Blood Anticoagulated with Ion-Balanced Heparin

Here, 1 tube (2 mL) of heparinized blood from radial artery will be collected for determining blood pH, lactate, partial oxygen pressure and arterial oxygen saturation before the administration of the first piperacillin/tazobactam infusion on each day of treatment. Analysis will be performed within 10 min after collection using an ABL800 Flex Blood gas analyzer (Radiometer Medical APS, Bronshoj, Denmark). Up to 5 of these samples will be collected from each participant and destroyed upon analysis.

#### 2.4.6. Sample ‘E’: Blood Anticoagulated with K_3_-EDTA

Next, 4 mL venous blood will be collected before the administration of the first piperacillin/tazobactam infusion on each day of treatment into a tube containing tripotassium ethylene diamine tetraacetate (K_3_-EDTA) to perform complete blood count analysis. Analysis will be performed within 4 h on Cell Dyn Sapphire and Cell Dyn Ruby analyzers (Abbott, Abbott Park, IL, USA). A maximum of 5 samples during the study will be obtained from each subject. Samples will be stored at 2–8 °C for 2 days following analysis.

#### 2.4.7. Sample ‘F’: Blood Anticoagulated with Sodium Citrate

Moreover, 2 mL of venous blood will be drawn into a tube containing sodium citrate once daily prior to initiating the first piperacillin/tazobactam infusion. After centrifugation with 2000 g, prothrombine time will be determined from plasma samples within 4 h on a Sysmex CS-2000i analyzer (Sysmex Hungary Ltd., Budapest, Hungary). A maximum of 5 samples will be obtained from each subject. Plasma samples will be stored at 2–8 °C for 2 days following analysis.

### 2.5. Disposal of Samples

At the end of the storage period, samples will be treated as hazardous samples and disposed of in accordance with the applicable regulations.

### 2.6. Protocols of Intervention

No intervention other than those performed as part of the treatment of recruited subjects guided according to the effective therapeutic protocols will be initiated. The collection of tracheal secretions as described here is part of the treatment. Blood samples will be drawn via a cannula inserted in the vena cava superior as a therapeutic measure. The collection of blood samples will not cause any discomfort or present any risks to subjects. Samples C, D and E are collected as described from all CAP patients receiving intensive care as part of their treatment. Samples B are going to be obtained for the purposes of the study.

### 2.7. Observed Parameters

Following admission to the ward, severity scores are going to be established to predict treatment outcomes, including simplified acute physiology score II (SAPS II), sequential organ failure assessment (SOFA), mortality probability model (MPM), confusion, yremia, respiratory rate, blood pressure, age ≥ 65 years system (CURB-65), and Pneumonia Severity Index (PSI) [28,29,30,31,32]. These scoring systems are components of the treatment protocol of the Central Department of Anesthesiology and Intensive Care Unit of Uzsoki Teaching Hospital. Demographic information (gender, age, race, height and body weight), general physical status, medical history (including detailed information on medications taken), circulatory parameters (systolic and diastolic blood pressure, mean arterial pressure, heart rate), the intensity of circulatory support, positive end-expiratory pressure, inspiratory fraction of oxygen, arterial partial pressure of oxygen and peripheral oxygen saturation will be recorded. Interviews with patients or, in case they are incapacitated, with relatives or next-of-kins, will be conducted to clarify their medical history. The posology of piperacillin/tazobactam, hydrocortisone and other medications, as well as the exact date and time of the beginning and the termination of each infusion, of the administration of hydrocortisone, and of the collection of tracheal secretions and blood samples, will be registered.

### 2.8. Data Evaluation

Data will be obtained from patient records (i.e., through observations and by performing calculations) and stored electronically in a spreadsheet format using Microsoft Excel (Microsoft Corporation, Redmond, WA, USA).

The minimum acceptable observational set is one complete set of daily samples. Additional issues corresponding to missing values will be addressed by applying the most suitable statistical strategy including, possibly, the exclusion of the variable, the imputation of the mean or median values or if inevitable the omission of the subject from the evaluation. Missing drug concentrations will not be replaced by calculated values.

**Figure 3:**
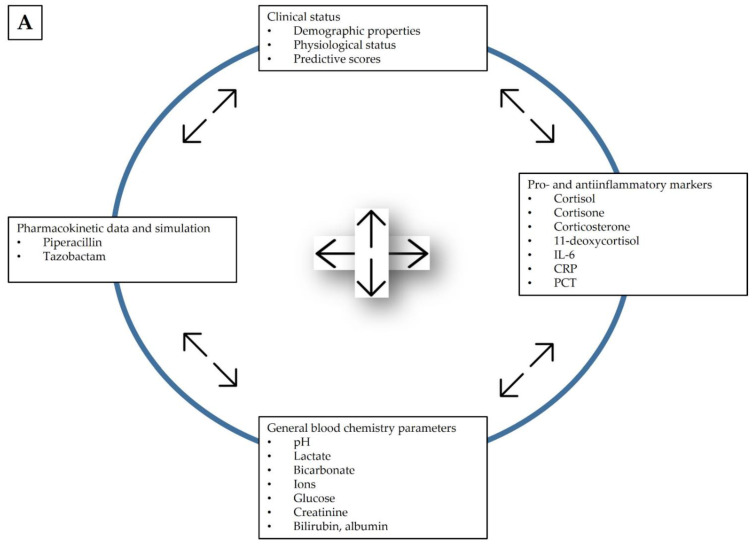

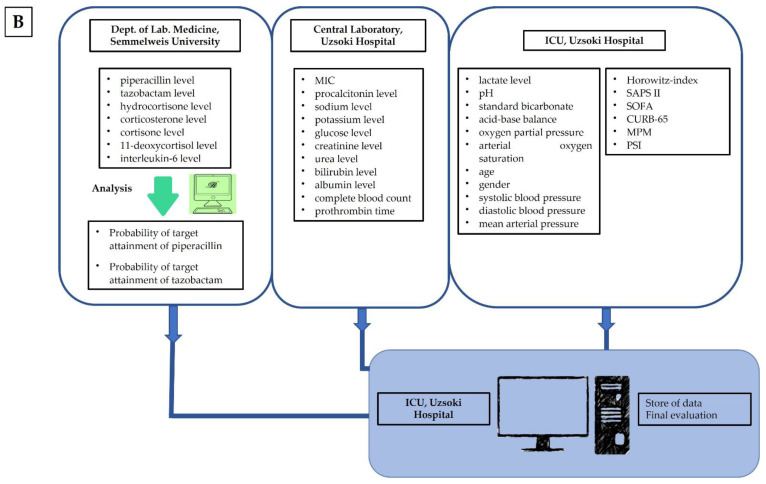
Measured parameters categorized by (**A**) study rationale and (**B**) the location of data collection, evaluation and storage. SAPS II simplified acute physiology score II; SOFA, sequential organ failure assessment; CRUB-65, confusion, uremia, respiratory rate, blood pressure, age > 65 years; MPM, mortality prediction model; PSI, patient safety indicators.

The different variables will belong to one of the following categories: primary or secondary outcome, descriptive, or predictive (Table 3). Primary outcomes are observed and calculated from data obtained by performing drug concentration measurements. Further, 5-day ICU mortality is also handled as a secondary outcome as it is an endpoint which depends on the levels of several other variables. General demographic information, physiological values and laboratory test results are handled as descriptive variables. Descriptive variables will be tested for inclusion as covariates in the population pharmacokinetic models to be constructed in the framework of the study. Finally, patient scores calculated by applying conventional scoring systems and employed to predict the chance of survival will be handled as predictive variables. A large number of parameters will be collected in various departments. Figure 3A shows the measured parameters categorized by the rationale of the study. The corresponding department of data collection, data storage and the place of final evaluation is summarized in Figure 3B.

Nonparametric statistical hypothesis tests will be applied to test the relationship between the values of descriptive and predictive variables, and primary and secondary outcomes. Due to the relatively small size of the cohort, this approach is expected to yield more valid results than those obtained using parametric tests following the transformation of data. Bi- or multivariate logistic regression analysis will be employed to create novel predictive scores based on the values of the observed and calculated variables. For each variable that remains in the model, the odds ratio will be calculated. Wald statistics will be employed to select the independent variables associated with the outcomes of interest. Receiver operating characteristic (ROC) curves of the predictive scores will be constructed to characterize their values in predicting the secondary outcome (5-day survival) [33]. The areas under the ROC curves will be calculated. The attainable sensitivities and the respective cut-off values will be determined.

Assay error equations of piperacillin and tazobactam are going to be constructed by calculating the standard deviations of measured concentrations in 3 independent experiments at a total of 20 spiked concentrations levels, and by using 20 independent serum matrices, which do not contain piperacillin or tazobactam, at each spiking level. Based on existing evidence, Theil’s regression will be performed on the concentration-standard deviation data pairs to establish the error equations [34,35].

Nonparametric population pharmacokinetic modeling and Monte-Carlo simulation are going to be conducted using PmetricsTM, a dedicated software package running in the R environment [36,37,38]. One- and two-compartment models will be explored. In addition to visual predictive checks, the evaluation of model performance and the comparison of rivaling models will use linear regression on observed versus predicted concentrations, shrinkage of random effects, correlating random effects, and by calculating the Akaike and the Bayesian information criteria, −2 × log-likelihoodg, the mean weighted squared prediction error and related descriptors. The approximation of the normal distribution of residuals is going to be assessed using the Kruskal–Wallis test. The statistical difference of the mean of residuals from zero is going to be calculated. Covariates will be investigated for inclusion in the models if the coefficient of the linear correlation between the candidate covariate and any of the random effects exceeds 0.8 (in case of continuous covariates), or if a statistically significant difference is found between the levels of the covariate and any of the random effects (in case of dichotomous and categorical covariates) at a significance level of 0.001.

### 2.9. Endpoints of the Observational Study

- **Primary endpoints:** (1) PK parameters of piperacillin and tazobactam and (2) the possible changes in concentrations of the monitored endogenous steroids during the five treatment days.
- **Secondary endpoints:** Based on Monte-Carlo simulation the probability target attainment (PTA) of piperacillin and tazobactam.
- **Tertiary endpoints:** the association between individual pharmacokinetic parameters of piperacillin and tazobactam and individual laboratory parameters that characterize physiological status.

### 2.10. Sample Size

Our work is intended to be a hypothesis-generating observational study yielding preliminary results that could be the basis of hypothesis-driven clinical investigations. In a report of CAP cases from 2016 to 2020 at Central Department of Anesthesiology and Intensive Care (Uzsoki Teaching Hospital, Budapest, Hungary) 122 patients with CAP were registered (30 patients per year). Wallenburg et al. conducted a clinical study with similar objectives in which work 39 patients were enrolled [39]. Based on the prevalence of CAP patients at Central Department of Anesthesiology and Intensive Care, as well as a study design of recent clinical researches our aim is to recruit 40 patients.

Additionally, the sample size was characterized to be suitable for considering the objectives of the study and with the intention to allow the formation of subpopulations during the evaluation of results.

### 2.11. Adverse Events

All therapeutic measures will be taken based on the clinical protocols applied at Uzsoki Teaching Hospital. Therefore, no specific adverse events other than the minimal discomfort caused by the collection of samples B are expected to occur.

## 3. Ethics and Dissemination

### 3.1. Ethical Considerations

The study was approved by the National Institute of Pharmacy and Nutrition (OGYÉI, Hungary) and by the National Scientific and Ethical Committee of the Medical Research Council (ETT-TUKEB, Hungary) (permission number: 261-IK/2020). The written informed consent of included subjects or, if they are incapacitated, of their relatives, will be obtained in advance. All information on subjects is treated anonymously for the purposes of the study. De-identification is performed by the study coordinator. The decision of participants or the persons acting on their behalf to exit the study will be effected immediately regardless of the underlying reason, and without any further adverse impact on their treatment. All provisions of the Declaration of Helsinki of the World Medical Association will be respected and complied with.

### 3.2. Score of Observations and Presentation Format

Standard scoring systems generally employed to describe the clinical condition and perspectives of subjects are going to be applied. Demographic data will be presented as medians and interquartile ranges. Pharmacokinetic model outputs, as well as the probabilities of target attainment are going to be provided in a graphical and tabular format as appropriate. To assess the relationship between candidate covariates and random effects, correlation plots and equations will be presented. The results of nonparametric hypothesis tests performed on unpaired data will be displayed along with boxplots. Covariate exploration will be conducted by comparing the performance of the respective hierarchical (nested) models.

### 3.3. Data Availability

The data that support the findings of this study will be openly available in Mendeley Data at doi:10.17632/w7gt8zdp3r.1.

### 3.4. Limitations

The first limitation of the planned study is that, given the fact that the relatively small number of patients admitted to ICU where the study will be conducted leaves little room for applying specific exclusion criteria. The clinical and physiological status of the included subjects is difficult to predict, consequently, the studied population is expected to be heterogeneous in terms of the studied variables. Second, the sampling and evaluation protocols are rather complex. Proper training and supervision of the personnel of the participating ICU will be executed to facilitate correct sample collection and documentation at the point of care.

Several samples and aliquots will be obtained from each subject which poses a major logistic challenge. We aim to overcome this difficulty by training the participating laboratory personnel on a regular basis (e.g., bimonthly) to assist them in performing their study-related tasks efficiently, by providing continuous supervision of the processes of the preanalytical phase by a laboratory professional, and by enforcing extensive documentation of all procedures.

Piperacillin and tazobactam are chemically unstable molecules, therefore, the proper preanalytical handling of samples B/1 is a key prerequisite for obtaining valid pharmacokinetic information. Training, supervision and extensive documentation of related processes will be affected carefully.

## 4. Discussion

To our knowledge, the planned study will be the first to characterize the relationship between the antibiotic and endogenous steroid concentrations during the administration of piperacillin/tazobactam and hydrocortisone to CAP patients treated at the ICU. Such therapies are associated with the high inter- and intra-individual variability of PK parameters [14], but are nevertheless employed empirically without pharmacokinetic considerations, and especially without understanding the relationship between antibiotic pharmacokinetics and steroid hormone status. Based on the findings of this hypothesis-generating study, further clinical investigations are expected to improve the efficacy of the intensive therapy of CAP patients.

## Figures and Tables

**Table 1:** Criteria of diagnosing community-acquired pneumonia [22].

Level of Significance	Description
Minor	Respiratory rate ≥ 30 breaths/min PaO_2_/FiO_2_ ratio ≤ 250 Multilobar infiltrates Confusion/disorientation Uremia (blood urea nitrogen (BUN)) level ≥ 7.14 mmol/L Leukopenia (white blood cell (WBC) count < 4 G/L) Thrombocytopenia (platelet count < 100 G/L) Hypothermia (core temperature < 36 °C) Hypotension requiring aggressive fluid resuscitation
Major	Invasive mechanical ventilation Septic shock with the need for vasopressors

**Table 2:** Laboratory values established in the participants. (A) Tracheal secretion; (B) and (C) tubes of venous native blood; (D) 1 tube of venous, heparinized blood; (E) 1 tube of venous, EDTA-treated blood; (F) 1 tube of venous blood treated with sodium citrate. BGA: blood gas analyzer, CA: clinical analyzer, HPLC-UV, high performance liquid chromatography with absorbance detection, INR: international normalized ratio, IST: in vitro susceptibility test, LC-MS/MS: liquid chromatography-tandem mass spectrometry, MIC: minimal inhibitory concentration.

Test	Method	Days of Sampling	Time of Sampling	Sample Identifier
MIC	IST (E-test)	1; 4	ordered by principal investigator	A
piperacillin level	HPLC-UV	1–5	3.25, 3.5, 4, 4.5, 5, and 5.5 h after administration of the 1st infusion of the day	B/1
tazobactam level	HPLC-UV	1–5	3.25, 3.5, 4, 4.5, 5, and 5.5 h after administration of the 1st infusion of the day	B/1
hydrocortisone (cortisol) level	LC-MS/MS	1–5	5.5 h after administration of the 1st infusion of the day	B/2
corticosterone level	LC-MS/MS	1–5	5.5 h after administration of the 1st infusion of the day	B/2
cortisone	LC-MS/MS	1–5	5.5 h after administration of the 1st infusion of the day	B/2
11-deoxycortisol level	LC-MS/MS	1–5	5.5 h after administration of the 1st infusion of the day	B/2
interleukin-6 level	CA	1–5	before administration of the 1st infusion of the day	C/1
procalcitonin level	CA	1–5	before administration of the 1st infusion of the day	C/2
sodium level	CA	1–5	before administration of the 1st infusion of the day	C/2
potassium level	CA	1–5	before administration of the 1st infusion of the day	C/2
glucose level	CA	1–5	before administration of the 1st infusion of the day	C/2
creatinine level	CA	1–5	before administration of the 1st infusion of the day	C/2
urea level	CA	1–5	before administration of the 1st infusion of the day	C/2
bilirubin level	CA	1–5	before administration of the 1st infusion of the day	C/2
albumin level	CA	1–5	before administration of the 1st infusion of the day	C/2
lactate level	BGA	1–5	ordered by principal investigator	D
pH	BGA	1–5	ordered by principal investigator	D
standard bicarbonate	BGA	1–5	ordered by principal investigator	D
acid-base balance	BGA	1–5	ordered by principal investigator	D
oxygen partial pressure	BGA	1–5	ordered by principal investigator	D
arterial oxygen saturation	BGA	1–5	ordered by principal investigator	D
complete blood count	hematology blood analyser	1–5	before administration of the 1st infusion of the day	E
prothrombin time	coagulometer	1–5	before administration of the 1st infusion of the day	F

**Table 3:** Variables considered for evaluation. SAPS II indicates simplified acute physiology score II; SOFA, sequential organ failure assessment; CRUB-65, confusion, uremia, respiratory rate, blood pressure, age > 65 years; MPM, mortality prediction model; PSI, patient safety indicators; PTA of piperacillin, probability of target attainment of piperacillin; PTA of tazobactam, probability of target attainment of tazobactam.

Variable	Category	Variable Type	Source/Method/Instrument/Stand
Age (Years)	Discrete	Descriptive	Patient record
Gender (Male/female)	Dichotomous	Descriptive	Patient record
Body mass index (kg m^−2^)	Continuous	Descriptive	Observation (height, weight) and calculation
Systolic blood pressure (mmHg)	Continuous	Descriptive	Observation
Diastolic blood pressure (mmHg)	Continuous	Descriptive	Observation
Mean arterial pressure (mmHg)	Continuous	Descriptive	Observation
Horowitz-index (no unit)	Continuous	Descriptive	Calculation
SAPS II (no unit)	Discrete	Predictive	Calculation
SOFA (no unit)	Discrete	Predictive	Calculation
CURB-65 (no unit)	Discrete	Predictive	Calculation
MPM (no unit)	Discrete	Predictive	Calculation
PSI (no unit)	Discrete	Predictive	Calculation
Serum IL-6 (pg mL^−1^)	Continuous	Descriptive	Observation (Laboratory measurement)
Procalcitonin (µg mL^−1^)	Continuous	Descriptive	Observation (Laboratory measurement)
Serum cortisol concentration (ng mL^−1^)	Continuous	Primary outcome	Observation (Laboratory measurement)
Serum cortisone concentration (ng mL^−1^)	Continuous	Primary outcome	Observation (Laboratory measurement)
Serum corticosterone concentration (ng mL^−1^)	Continuous	Primary outcome	Observation (Laboratory measurement)
Serum 11-deoxicortisol concentration (ng mL^−1^)	Continuous	Primary outcome	Observation (Laboratory measurement)
PTA of piperacillin (%)	Complex	Primary outcome	Observation (piperacillin concentration, minimal inhibitory concentration) and calculation
PTA of tazobactam (%)	Complex	Primary outcome	Observation (tazobactam concentration, minimal inhibitory concentration) and calculation
5-day ICU mortality (%)	Continuous	Secondary outcome	Observation
Length of period without ventilation (Days)	Discrete	Descriptive	Observation
Length of catecholamine treatment-free period (Days)	Discrete	Descriptive	Observation
Length of insulin therapy (Days)	Discrete	Descriptive	Observation

## Data Availability

The data that support the findings of this study will be openly available in Mendeley Data at doi:10.17632/w7gt8zdp3r.1.

## Supplementary Materials

The following supporting information can be downloaded at: https://www.mdpi.com/article/10.3390/jcm11144140/s1, File S1 (PDF): STROBE Statement—Checklist of items that should be included in reports of cohort studies.
